# Conveying misinformation: Top-ranked Japanese books on tobacco

**DOI:** 10.1186/1617-9625-9-3

**Published:** 2011-01-24

**Authors:** Yuko Kanamori, Ruth E Malone

**Affiliations:** 1 Independent Consultant, Bryan, TX 77802 USA; 2 Department of Social & Behavioral Sciences, 3333 California St, Suite 455, University of California, San Francisco, San Francisco, CA 94118, USA

## Abstract

**Background:**

Tobacco control efforts in Japan have lagged other high income countries, possibly because the Japanese government partially owns Japan Tobacco, Inc. In Japan, tobacco use is still often regarded as an issue of manners rather than an issue of health. Information about tobacco is available, but may not always be accurate. We explored what information Japanese consumers might access by reading popular Japanese books about tobacco.

**Methods:**

We searched Amazon.com Japan using the term "Tobacco", identifying the top 12 books by "relevance" and "bestselling." We eliminated duplicates and books not concerned with tobacco use and classified the remaining books as pro-smoking, anti-smoking, or neutral. We reviewed the pro-smoking books, published 2004-2009, and analyzed examples of misinformation by theme.

**Results:**

Pro-smoking popular books conveyed five types of misinformation: doubt about science; suggestions that smoking increased health, longevity, virility, etc.; trivializing tobacco's effects; attacking public health advocates/authorities; and linking tobacco use with authenticity, history, or civil rights. At least one book was authored by a former Japan Tobacco employee; another used a popular Japan Tobacco advertising phrase.

**Conclusions:**

Creating doubt and confusion about tobacco serves tobacco industry interests and re-creates a strategy developed by US tobacco interests more than 40 years ago. Japanese readers may be misled by texts such as those reviewed. Tobacco control and public health advocates in Japan and globally should expose and counter such misinformation. "Naming and shaming" may be effective.

## Introduction

Since it is established that smoking tobacco causes disease [1,2], and the landmark Japanese study showing that secondhand smoke (SHS) causes disease in nonsmokers was published almost 30 years ago [3], it is surprising that tobacco use in Japan remains so widespread that the country has been called a smokers' paradise [4]. Although nationally, smoking rates have continued dropping [5], 39.4% of Japanese men and 11.0% of Japanese women still smoke [5], and 24% of health professionals are smokers, compared with 4% overall among U.S. health professionals [6]. Tobacco control efforts in Japan lag many other countries, possibly because the Japanese government is the majority owner of Japan Tobacco, Inc. (JT) [7]. Given an absence until recently of effective central government action, it is unclear why smoking has dropped, but rates are still higher than those in many higher-income countries.

The Tobacco Industries Act protects JT's business; the government is required to retain least 50% of JT's stock [8,9]. While the government seeks to profit from tobacco sales, the Ministry of Health, Labor and Welfare (MHLW) is concerned with protecting health. The government, then, is conflicted; the result has been (until very recently) "a wordless annihilation of much meaningful tobacco control program development at the national government level [9,10]."

The social and informational context in Japan means that tobacco use is still often regarded more as an issue of good manners than a health threat [10,11]. Information about tobacco is available from many sources, but may not always be complete or accurate. Because popular books are a way to gain information about any topic, and the website Amazon.com has become a common way to identify and order books, we examined books available on the Japanese Amazon.com website to appraise their messages about tobacco.

## Methods

In July 2009, we searched for books using the keyword "Tobacco" (たばこ) on Amazon Japan (amazon.co.jp). We compared the top 12 books sorted by "relevance" and the top 12 by "bestselling". (The website provides no definition of "relevance" or "bestselling.") After eliminating duplicates, the remaining 19 books were purchased and reviewed by the first author, a native Japanese speaker. Two books were excluded because they did not discuss smoking, even though the word tobacco was in the title. The remaining books were classified as pro-smoking, anti-smoking, or neutral. A pro-smoking book was defined as any book generally supporting smoking cigarettes, or challenging the idea that smoking was unhealthy. An anti-smoking book was defined as emphasizing that smoking was unhealthy. Neutral books covered the history of tobacco and/or did not take a position on smoking.

Pro-smoking books were reviewed for content and broadly representative excerpts selected, focusing on examples of misinformation about smoking. Draft English translations were prepared by the first author and reviewed and categorized by both authors. A consultant who majored in teaching Japanese and frequently speaks English and Japanese checked and refined the draft translations by comparing them against the original Japanese in the books. Examples were summarized by theme to illustrate the types of misinformation a Japanese reader might encounter.

### Limitations

We examined a small number of books, which may not be representative of the population of books actually read by Japanese citizens interested in tobacco. Other search terms might retrieve a different set of books. Lists change; we do not know how often they are updated or how long these books remained listed. Nevertheless, it seems reasonable that someone not yet knowledgeable about tobacco might search using this word, and that these would be books most likely to be seen (and in the case of bestsellers, purchased) by Amazon's Japanese users.

We were unable to do a similar study examining in detail books on the Amazon site in other countries, but on Feb 18 2010, we examined the top 12 books by bestselling and relevance on the United States Amazon site, searching using the word "tobacco." Examining titles and descriptions of the books, we found only two of 24 unique listed books that appeared pro-smoking: a guide to cigars, #9 by bestselling, and the book *The health benefits of tobacco*, by William Campbell Douglass, #3 on the relevance list. Dr. Douglass is a well-known 'maverick' who apparently makes a living claiming to debunk so-called health and medical 'myths' (generally without offering scientific evidence for his claims) and selling books and supplements online. He also claims the Internal Revenue Service is unconstitutional and apparently has argued that paying taxes is treasonous because, he claims, there are communists in the government [12]. In any case, the U.S. site appeared to feature a far smaller proportion of books with misleading material than appeared on the Japanese version of the site. Selected quotations, while broadly representative of book content, were not randomly chosen, but were selected to illustrate misinformation conveyed. We did not have access to reader data, so we can only theorize about how reading these books might influence smoking beliefs and behaviors.

## Results

Of the 17 books, 9 were pro-smoking, 7 anti-smoking, and 1 neutral. By relevance, 7 of the top 12 books (58%) were pro-smoking, as were 4 of the top 12 (36%) bestsellers. Of 7 pro-smoking books on the relevance list, two were also bestsellers. All anti-smoking books on the relevance list mentioned how tobacco companies manipulated the public. No smoking cessation books appeared on the relevance list; 4 of 6 anti-smoking books on the bestselling list focused on cessation.

### Pro-smoking books

Nine pro-smoking books were reviewed. We sampled material from five of these (Table 1) to illustrate the types of misinformation conveyed. Among the other four pro-tobacco books reviewed, one was a guide to cigars, one was a pictorial history of tobacco packaging, and two were graphic cartoon books about smokers' manners, published by Japan Tobacco, Inc. [13-16]. The latter used stylized cartoons to suggest, among other things, that smokers should buy cigarettes and portable ashtrays before going to summer festivals, or take a break by smoking after perspiring while dancing, and appeared intended to reinforce smoking as a normal part of social events, while minimizing social conflicts [15].

**Table 1:** Misinformation Themes

Theme categories	Examples
**1. Creating doubt about science**	As stated by BAT and JT, it is difficult to scientifically assess the effect of secondhand smoke in chronic illnesses. The current assessment of the effect of secondhand smoke is overrated. ^19 ^(p34)

	There is no correlation between the percentage of smokers/amount of smoking and the average life expectancy when looking at data from various countries. ^19 ^(p75)

	Many epidemiological study results have revealed that smokers, in comparison to non-smokers, have higher risk of death through contracting various diseases and that they have shorter average life expectancy by 10 years. However, the question is how did Japanese males, who are known to be the world's greatest tobacco lovers, prolong their life expectancy rapidly and become the world's number one in both average life expectancy and healthy life expectancy? They are also expected to continue to maintain this status. In other words, there is a contradiction that is hard to explain between people's life expectancy (the greatest statistical data) and the results of epidemiological study. ^19 ^(p84)

	There are certain conditions that must be met in order to say that smoking causes lung cancer. Although lung cancer caused by smoking is different from cancers caused by viral infection, it is the same in a sense that they are both exogenous diseases. If the following three conditions are met, everyone will be convinced that smoking does cause lung cancer. 1. All lung cancer patients are smokers. 2. Non-smokers will never be diagnosed with lung cancer. 3. Create lung cancer in animals by putting them in the same condition/situation as smokers. ^21 ^(p62)

	It's well-known that tobacco is toxic, but there is a significant lack of scientific evidence for this fact. ^22 ^(p8)

	To say that secondhand smoke causes "early death" is merely a myth. ^22 ^(p10)

	Most data that presents the danger in smoking is based on epidemiological research, which used statistics to project the cause of illnesses related to smoking, but there is very little data from pathological research. ^22 ^(p34)

	As Japan Tobacco Inc. (JT) states, the correlation between tobacco and cancer is not yet fully clear. Therefore, as journalists, one should report/write from a more objective standpoint. ^22 ^(p63)

	Tobacco, along with alcohol, has been widely popular amongst people for over 400 years. Massive human experimentation (pm how tobacco affects the human body) has already been done. Even if tobacco were dangerous, how harmful could it be? ^22 ^(p63)

	Aside from special cases, breathing in secondhand smoke is probably unproblematic. ^22 ^(p136)

	The fact that secondhand smoke causes various diseases is also inaccurate. ^23 ^(p102)

	Unlike smokers, non-smokers are not fully inhaling the smoke to the deep ends of the lung, so in actuality, the phenomenon of secondhand smoke does not exist. Even if there were secondhand smoke, it could simple be taken care of by opening the window or turning on the fan. Being affected by secondhand smoke in the outdoors is impossible. ^23 ^(p124)

	Through epidemiological study, scientists may have found that there are carcinogens in cigarettes. However, the theory that directly attributes the cause of cancer to smoking is pure imagination. No matter how much emphasis is put on the results of epidemiological studies, or the fact that over 40 kinds of carcinogens are found in cigarettes, this still does not scientifically prove that smoking causes cancer. This is just more circumstantial evidence saying tobacco is "suspicious" (believed to cause cancer). ^24 ^(p83)

	The anti-smoking atmosphere has escalated to the current state because the entire world, including a powerful organization like the WHO, believes smoking is hazardous without any evidence. ^24 ^(p88-89)

**2. Suggesting that smoking increases health, longevity, virility, coping, happiness**	There are people who are able to maintain their health and prolong their life expectancy ("exceeding survivors") through moderate smoking. ^19 ^(p85)

	It is clear that elderly people who love to smoke in nursing homes live much longer. ^19 ^(p153)

	Smoking causes one to calm down, and allows him to handle situations better. ^21 ^(p21)

	If you can figure out what your source of happiness is, you can be healthy and still enjoy your cigarette. ^21 ^(p220)

	If tobacco really is as hazardous as it is said to be, how does Japan have the longest life expectancy even with the highest smoking rate in the world? ^22 ^(p9)

	Smokers can be just as health as nonsmokers, or even healthier, as long as they keep a healthy diet and live a low-stress life. ^22 ^(p107)

	There is data that states the life expectancy of those who smoke pipes or cigars is just as long as, or even longer than, non-smokers. ^23 ^(p92)

	Most importantly, tobacco is a tasty, precious gift from God for us (the smokers) to lead a better life. ^23 ^(p181)

	As the rate of smoking among Japanese men decreases, so has the birth rate. ^24 ^(p15)

	One way of judging a man is through his work ability, and men who smoke are historically viewed to be good at their jobs, because nicotine's pharmacological effect works advantageously on one's work capacity. Therefore, tobacco itself has become a status symbol for men. ^24 ^(p38)

	Nicotine is a powerful weapon for men to achieve better status. However, it is pitiful that men, who have once experienced the powerful effect of nicotine, quit smoking. ^24 ^(p72)

	Men who quit smoking are not only losing its medical value, but are also viewed as men of low ambition, men who have let go of their masculinity. ^24 ^(p118-119)

**3. Trivializing the effects of tobacco use**	Consuming tobacco is troublesome (because of our gag reflex), but accidentally inhaling peanuts (through the bronchial tube) is even more troublesome. ^21 ^(p92)

	There are many cancer-causing substances in the world. The only problem with tobacco is the manner of smokers. ^22 ^(p8)

	Issues in tobacco can be solved if smoking manners are reviewed. ^22^(p19)

	If you are not around smokers, you are not subjected to secondhand smoke, but you breathe is polluted air (exhaust from cars and factories) whether you like it or not. ^22 ^(p64)

	Anti-tobacco activists are obsessed over secondhand smoke containing more carbon monoxide than firsthand smoke, but there is no need to worry about that. The amount of carbon monoxide which tobacco contains is very small, plus carbon monoxide coverts into carbon dioxide in the atmosphere. ^22 ^(p103)

	Addiction to tobacco is not as powerful as addition to narcotic drugs. ^22 ^(p114)

	Considering that all human beings die, it is questionable to single out and harass the smokers by addressing only the harm tobacco may possess. ^23^(p76)

	Tobacco gives happiness and pleasure to the lives of those who smoke, just like how coffee, tea, and alcohol are enjoyed by many people. ^23^(p88)

**4. Attacking public health advocates and authorities as liars and paternalists**	The warning "Be careful with excessive smoking for your health" on a cigarette package is a typical example of paternalism. ^19 ^(p53)

	When providing that tobacco is hazardous, pictures of a black, discolored lung are frequently used. These pictures are complexly false. ^21 ^(p4)

	Cigarette smoke is particles of moisture vapor and tar, so it does not accumulate in the lungs. Hence, the blackening of lungs of heavy smokers is a lie. ^21^(p66)

	Quite frankly, what these anti-smoking activists are doing seems like the beginning of a folly that hearkens back to things human beings have caused in the past, such as indulgences sold by the Catholic church, the witch hunt, prohibition, slavery, the holocaust, discrimination, war, and more. ^22 ^(p20)

	If the state and federal government win a lawsuit against tobacco companies, a portion of reparations is said to go to the World Health Organization. What the WHO is up to is not to abolish tobacco entirely, but actually to team up with governments from around the world to make profit from lawsuits against the tobacco industry. ^22 ^(p32)

	These anti-smoking activities are scientifically, socially, and culturally doing no good to the world. ^22 ^(p127)

	There is not enough science-based evidence to prove that smoking is the main cause of cancers, or to say that it is scientifically "evident" that tobacco causes cancer. For this, organizations like the WHO and other anti-smoking groups are nothing but liars. ^23 ^(p79)

	It is probably true that smoking has smoke effect on a human body. However, it is also true that many of the numbers derived from these studies are exaggerated. (For example,) the fact that 90% of lung cancer cases are caused by smoking is clearly a fabrication. ^23 ^(p84)

	Anti-smokers are the greatest evils. Adolf Hitler is known for disliking tobacco. ^24) ^(p154, 156)

**5. Linking tobacco with authenticity, history, or civil rights**	I support smoking, because I believe it is a unique gift of pleasure from the Native Americans, a culture that was developed through a long period of history. ^23 ^(p70)

	Recently, even hospitals are becoming smoke-free. Unlike schools and work places, hospitals, for some people, are a place where they will spend the end of their lives. It is humanely wrong to forcefully take away what may be some patients' only enjoyment. Even those who face execution in the prison are allowed to smoke for the last time. Yet, patients who await their death in a hospital cannot have a simple enjoyment as a cigarette. ^23 ^(p89)

	Smoking cigarettes is a ritual for peace among Native Americans. ^24 ^(p158)

The 5 books excerpted were all published since 2006; all authors noted that they were smokers and books featured similar content. While suggesting that smoking cigarettes was not harmless, they put far greater emphasis on the social goods of smoking, arguing that tobacco was not harmful enough to require regulation. They emphasized Japan's long life expectancies compared to other industrialized countries despite Japan's higher smoking rates, ignoring likely contributions of the Japanese diet and other factors to this phenomenon. The books suggested that little scientific data proved that tobacco was harmful, and that other substances were worse.

The authors consistently portrayed smoking as a personal "choice" or preference, an issue of "rights" and "freedom," and said government should not get involved. If there was a tobacco problem, according to these authors, it was smokers' manners, not the product. They also linked smoking with Native American peacemaking ceremonies. Some of their assertions exactly match JT's claims [17,18], such as blaming smokers' manners for any problems. It is unknown whether tobacco companies sponsored these publications.

Below, we provide brief descriptions of the five pro-smoking books reviewed:

### The lie behind the fact, "Cigarettes: 100 harms and zero benefit" (2007)[19]

This pro-smoking book by Takeda, second by relevance and 18^th ^among bestsellers, argued that SHS effects on nonsmokers were exaggerated. The author worked for Japan Tobacco until 1995; his argument echoes JT's SHS claims [20]. He also claimed, without citing evidence: "it is clear that elderly people in nursing homes who love to smoke live much longer" (p. 153).

### Objection! To the theory of harmful tobacco (2006) [21]

Oncologist Haruhiko Natori and sociologist Masayuki Uesugi coauthored this book, 7^th ^by relevance and 16^th ^among bestselling. Among other things, it sought to convince readers that tobacco was not harmful by equating smoking with other behavior that might harm human health too, such as accidentally inhaling peanuts. Use of JT's well-known advertising phrase, "I feel great, and cigarettes taste great!" ("今日も元気だ、たばこがうまい") suggested a JT connection.

### Tobacco is nutrition if you are a smoker (2007)[22]

Fumiaki Matsugae, lawyer and journalist, authored this book, ninth by relevance. Matsugae admitted that tobacco is harmful, but asserted "a significant lack of scientific evidence" (p. 9) for tobacco's toxicity. Like Takeda, he questioned, "if tobacco really is as hazardous as it is said to be, how do the Japanese have the longest life expectancy even with the highest smoking rate in the world?" (p. 9).

### Tobacco Hunting (2009) [23]

Authored by journalist and university professor Hisashi Muroi, this was eleventh by relevance and third among bestselling; the author described smoking as "a unique gift of pleasure" (p. 70). Since people are mortal, he argued, there was no point in trying to discourage smoking: "considering that all human beings die, it is questionable to single out and harass smokers by addressing only the harm tobacco may possess" (p. 76).

### Tobacco is God's gift (2008) [24]

Physician author Akira Hashiuchi claimed a correlation between smoking and birthrate suggests that smoking actually increases the birthrate. He also claimed smoking was socially advantageous for men, arguing that "smokers have higher social status" (p. 38). Former smokers were "men who have let go of their masculinity" (pp.118-119). Non-smoking was associated with evil because, he argued, Hitler hated smoking.

## Discussion

Japanese readers who use the Amazon.co.jp website to search for books about tobacco may be misled by texts such as these. While our search also retrieved anti-smoking books, the positioning of these pro-smoking authors as "scientific" or "medical" authorities claiming that smoking was more beneficial than harmful could influence beliefs and decisions about tobacco use and the desirability of smoking cessation. Public health advocates in Japan (and globally) should expose and counter such misinformation.

More than 40 years ago, a U.S. tobacco company internal document asserted: "Doubt is our product since it is the best means of competing with the "body of fact" that exists in the mind of the general public. It is also the means of establishing a controversy [25]." While we lack direct evidence that JT influenced these books, at least one was authored by a former JT employee, another uses a well-known JT advertising slogan, and several echo JT claims, suggesting continuation of a long-discredited strategy first perfected by U.S. tobacco companies.

There are, however, hopeful signs. Kanagawa prefecture recently implemented the nation's first smoke-free restaurant ordinance [4]. In February 2010, the Ministry of Health, Labor and Welfare issued "guidelines" on smoke-free restaurants and workplaces [26] and made clear that the disease effects of SHS have been scientifically established [27]. In addition, in October 2010, the tobacco tax was increased, raising the price of a pack of cigarettes by approximately $1. While cigarette sales in September rose 88% over a year ago due to last-minute buying to stock up [28], it appears that many smokers decided to quit smoking due to the tax increase; tobacco sales declined in October after the increase [29], and demand for antismoking medications is reportedly higher than expected [30]. Perhaps these developments, and continued global efforts to denormalize smoking, will help counter misleading claims made in books such as those we reviewed. Meanwhile, tobacco control advocates in Japan may wish to "name and shame" those authors who promulgate such misinformation.

## Competing interests

The authors declare no competing interests. REM owns 1 share apiece of stock in Philip Morris USA, PM International, and Reynolds American tobacco companies for research and advocacy purposes.

## Authors' contributions

YK originated the idea for the study, collected and analyzed data, did first translations, and wrote the first draft of the paper. REM supervised the study, analyzed data and designed the conceptual matrix, and participated in writing and editing all drafts. Both authors read and approved the final manuscript.
